# PEMOCS: theory derivation of a concept for PErsonalized MOtor-Cognitive exergame training in chronic Stroke—a methodological paper with an application example

**DOI:** 10.3389/fspor.2024.1397949

**Published:** 2024-06-10

**Authors:** Simone K. Huber, Patrick Manser, Eling D. de Bruin

**Affiliations:** ^1^ Physiotherapy and Occupational Therapy Research Centre, Directorate of Research and Education, University Hospital Zurich, Zurich, Switzerland; ^2^ Motor Control and Learning Group, Institute of Human Movement Sciences and Sport, Department of Health Sciences and Technology, ETH Zurich, Zurich, Switzerland; ^3^ Department of Health, OST—Eastern Swiss University of Applied Sciences, St. Gallen, Switzerland; ^4^ Division of Physiotherapy, Department of Neurobiology, Care Sciences and Society, Karolinska Institute, Stockholm, Sweden

**Keywords:** motor-cognitive training, exergames, virtual reality, personalization, user-centered technology, neuroplasticity, motor learning, stroke

## Abstract

**Background:**

Coping with residual cognitive and gait impairments is a prominent unmet need in community-dwelling chronic stroke survivors. Motor-cognitive exergames may be promising to address this unmet need. However, many studies have so far implemented motor-cognitive exergame interventions in an unstructured manner and suitable application protocols remain yet unclear. We, therefore, aimed to summarize existing literature on this topic, and developed a training concept for motor-cognitive exergame interventions in chronic stroke.

**Methods:**

The development of the training concept for personalized motor-cognitive exergame training for stroke (PEMOCS) followed Theory Derivation procedures. This comprised (1.1) a thorough (narrative) literature search on long-term stroke rehabilitation; (1.2) a wider literature search beyond the topic of interest to identify analogies, and to induce creativity; (2) the identification of parent theories; (3) the adoption of suitable content or structure of the main parent theory; and (4) the induction of modifications to adapt it to the new field of interest. We also considered several aspects of the “Framework for Developing and Evaluating Complex Interventions” by the Medical Research Council. Specifically, a feasibility study was conducted, and refining actions based on the findings were performed.

**Results:**

A training concept for improving cognitive functions and gait in community-dwelling chronic stroke survivors should consider the principles for neuroplasticity, (motor) skill learning, and training. We suggest using a step-based exergame training for at least 12 weeks, 2–3 times a week for approximately 45 min. Gentile's Taxonomy for Motor Learning was identified as suitable fundament for the personalized progression and variability rules, and extended by a third cognitive dimension. Concepts and models from related fields inspired further additions and modifications to the concept.

**Conclusion:**

We propose the PEMOCS concept for improving cognitive functioning and gait in community-dwelling chronic stroke survivors, which serves as a guide for structuring and implementing motor-cognitive exergame interventions. Future research should focus on developing objective performance parameters that enable personalized progression independent of the chosen exergame type.

## 1 Introduction

Stroke and its consequences are a serious public health challenge worldwide. In 2019, stroke was the third-most cause for disability-adjusted life years (DALYs) (1). Stroke can cause serious motor and cognitive impairments (2), which reside in the long-term in approximately two thirds of stroke survivors, potentially leaving them with impaired daily-life functioning and reduced health-related quality of life (3, 4). Accordingly, coping with long-term consequences and impairments is an important research priority of stroke survivors, even if they regained living in the community (5, 6). Moreover, stroke survivors repeatedly report unmet needs including that they lack support by health-care systems, continuous therapy, and services for secondary prevention (3, 7, 8).

Especially cognitive impairments have been overlooked and neglected in stroke rehabilitation for a long time (9, 10). Cognitive deficits after stroke are a key determinant of the long-term outcome of patients and associated with mortality, dependency, and depression within five years post-stroke (11). Notably, cognitive deficits are also highly prevalent in patients with seemingly good clinical outcome and few physical impairments (9, 12). In line with this, stroke survivors and their care-givers reported that “Improving Cognition” was their top research priority (5). Until today, community-dwelling chronic stroke survivors name the treatment of cognitive deficits to be one of their most common unmet needs (3, 8) and request research in this area (6). This need is mirrored in a recent scientific statement from the American Heart Association/American Stroke Association (13). Nevertheless, clear recommendations for cognitive rehabilitation after stroke are currently missing (14).

In motoric cognitive risk syndrome after stroke, cognitive impairments have recently been linked to impairments in gait ability (15). Evidence suggests that cognitive functions and gait share structural and functional roots within the central nervous system and may improve but also decline in a collective way (16–18). It is, therefore, not surprising that also impairments related to walking, mobility and balance are consistently high on stroke survivors' list of research priorities (5, 6). It may, therefore, be beneficial to combine motor and cognitive training to target the two inter-twined systems (16, 19). Evidence exists from healthy older adult populations that both, cognitive functions and gait, may additionally benefit from combined cognitive-physical interventions compared to cognitive or physical trainings alone (20–25). Additionally, first systematic investigations have been performed in (chronic) stroke populations and a superior effect of combined interventions on gait was observed (26–28). However, the effects on cognitive functions remain unclear due to a lack of studies investigating the effect of motor-cognitive training on cognitive functions in stroke (28, 29).

A specific type of motor-cognitive training are exergames, video games that require the trainee to be physically active for playing the game (30). Exergames have been found to improve functional outcomes in chronic stroke (31), as well as motor functions including balance and gait in healthy older adults and neurological populations (28, 32–40). Exergames have also been found to improve cognitive functions in healthy older adults, neurological and general populations (41–47). Therefore, motor-cognitive exergames have been suggested as an adjunct to usual care and as a strategy to avoid deconditioning when therapy is discontinued (35, 42). In our systematic review, we proposed that motor-cognitive exergame training may be the most effective type of motor-cognitive training for improving gait and cognitive functions in chronic stroke (28). However, we also found that more studies on motor-cognitive (exergame) training in chronic stroke are needed. Future studies should be based on a theoretical rationale since large heterogeneity is currently observed. This observed heterogeneity may be explained by the wide range of intervention protocols that are applied in past studies, with missing information on likely important intervention details in these publications (28). Study reports rarely contained a clear rationale justifying the used training system and applied training variables; for example total volume, frequency, or intensity of training. Furthermore, information on applied progression or variation is often completely missing (28).

In rehabilitation, a “*one-fits-all*” approach is insufficient to meet the needs of each individual patient (48, 49). To make rehabilitation interventions successful, it is important to consider that not all participating individuals will be able to train at the same absolute intensity, progress in the same time course, or prefer the same activities. This implies that the interventions should be personalized and tailored to each individual by considering their personal abilities and impairment levels (50, 51). Personalization protocols are a further “missing piece” in many training studies, or in case considered, not described with sufficient details in existing publications. It is, therefore, unclear what a minimum dosage of motor-cognitive exergame training for improving cognitive functions and gait in chronic stroke should be, and how the interventions should be personally progressed and varied (28, 42).

Therefore, the aim of this methodological paper was to narratively summarize existing literature on the topic and, based on that, develop a theoretical concept for personalized motor-cognitive exergame training for stroke (PEMOCS) with the aim to improve cognitive functions and gait in chronic stroke survivors. During that, we aimed to elaborate recommendations for an effective type and dosage of motor-cognitive exergame training in this context.

## 2 Materials and methods

For the developmental process of our concept for personalized motor-cognitive exergame training in **s**troke (PEMOCS) we followed Walker and Avant's Theory Derivation procedures (52). Theory Derivation uses analogy in explanations and predictions of phenomena in different fields to develop new concepts. It is a “*creative and focused way to develop theory in a new field*” (52). Theory Derivation is useful if several existing concepts seem relevant for the development of a new concept; however, their relationship is yet unclear. Analogy with relationships in another field can help induce modifications to the existing theory to adapt it to the new field of interest (52). Therefore, Theory Derivation is an iterative process, where one goes forth and back in steps until the striven theory is accomplished. Based on these basic steps for Theory Derivation presented in (52), we defined the following steps to accomplish our goal.

(1.1) Literature Search 1: We started with a thorough narrative literature research on stroke rehabilitation with a special focus on long-term interventions for community-dwelling stroke survivors. We aimed to identify general principles for stroke rehabilitation, which are summarized in the first result section “Core Principles”. By combining these core principles, we aimed to define, which “Components” the PEMOCS concept should consist of.
(1.2) Literature Search 2: To achieve sophisticated knowledge beyond the topic of interest, which is essential in Theory Derivation for analogies to be discovered and creativity to be induced, we widened our narrative literature research to related topics and fields. Guided by the “Core Principles” identified in literature search 1, we gathered evidence on motor-cognitive and exergame interventions in other populations (i.e., healthy older adults, other neurological populations, …), as well as related interventions (i.e., physical training, cognitive rehabilitation, …). This narrative search was repeated at regular time intervals throughout the developmental process to catch latest publications.
(2) Parent Theories: To start the Theory Derivation process, we searched for parent theories. We intended to find one main parent theory, which should be a suitable parent framework to build the base for a structured implementation of motor-cognitive interventions for improving cognitive functioning and gait in chronic stroke. Further existing theories and concepts in the field of the defined “Core Principles” were searched to guide the adaption of this framework to the new setting.
(3) What to Keep: Next, we identified what content/structure from the main parent theory was to be used for the new theory. To do so, the specific analogies of the parent theory and the phenomenon of interest were discovered. We, therefore, studied the chosen main parent theory in detail, and identified its content, which suits the topics motor-cognitive exergame interventions, cognitive functioning, gait, and chronic stroke.
(4) What to Add and Change: Theory Derivation always requires modifications to the parent theory to derive a new theory. Therefore, we used our gained knowledge from step 1 to adapt the parent framework and integrate content from the other parent theories from step 2 into the new theory. This included identifying guidelines, recommendations, and evidence on how to implement motor-cognitive exergame trainings for improving cognitive functions and gait in chronic stroke.

During these five steps, we also considered several aspects of the “Framework for Developing and Evaluating Complex Interventions” by the Medical Research Council (53). Specifically, a feasibility study with a first draft of the concept was conducted, which delivered further insights into practical issues of the concept and enabled further redefinition (54). The refining actions that were performed after this feasibility study are described in the last result section “3.5 Refining Steps after Feasibility Study”. As a result, we describe the PEMOCS concept in section “3.4 What to Change and Add: the PEMOCS Concept”, present action steps on how to implement it in every sub-section (Table 1), and make an application example (Supplementary S1). Overall, the developmental process was performed from September 2019 until August 2022. The effects of the PEMOCS concept, as worked out in the application example, on cognitive functions and gait in chronic stroke are currently evaluated in a randomized controlled trial [(58), clinicaltrials.gov, NCT05524727].

**Table 1:** Overview of the final PEMOCS concept with action steps to follow for applying it.

Section		Action steps	Example
1	Exergame Type	Choose a type of motor-cognitive exergame training based on recommendations, reasoning, accessibility, practical applicability, and preference of the target group.	Supplementary S1, Section S1
2	Training Dosage	Define the exercise variables that will be applied: total volume, frequency, density, session time, intensity. Consider evidence based recommendations as well as practical aspects such as how often and how long participants can train, if the session should be supervised a.s.f.	Supplementary S1, Section S2 and Supplementary Table S1
3.0	Cognitive Dimension	(a) Determine, how many and which cognitive domains are targeted by the chosen intervention. From this, define the number of the cognitive sub-dimensions. (b) Prepare the extended taxonomy with the determined number of cognitive sub-dimensions.	Figure 1 and Supplementary S1, Section S3
3.1	Personalizing the allocation of the cognitive sub-dimensions	(a) Choose suitable neuropsychological assessments that measure the identified cognitive functions. (b) Define how the order from most to least impaired domain will be determined at baseline.	Supplementary S1, Section S3.1 and Supplementary Table S2
3.2	Assigning motor-cognitive tasks to the sub-dimensions of the extended taxonomy	(a) Make a list of all motor-cognitive activities and versions thereof that will be performed in the chosen motor-cognitive exergame intervention. (b) Allocate all these activities to one of the motor-cognitive skill categories in the prepared extended taxonomy. If many skill categories remain empty, try to establish additional versions to fill the gaps.	Supplementary S2 and Supplementary Table S5 Supplementary S1, Supplementary Figure S1
3.3	Progression from session to session	(a) Check if the suggested difficulty levels apply well with all other so far determined components. If necessary, fuse some levels or split into sub-levels. (b) Choose an appropriate objective measure of performance suiting the chosen motor-cognitive exergame intervention. (c) Determine how the objective and subjective measures of the participant's challenge will be combined to derive the progression steps.	Supplementary S1, Section S3.3 including “Addition: Sublevels” & Supplementary Figures S2, S3
3.4	Progression within each session	(a) Determine how the task difficulty curve will be applied over the blocks to ensure the peak of task difficulty in the middle part of the session. (b) Considering the number of cognitive sub-dimensions, determine the order in which the different domains will be trained in each block. Ensure the increase, peak, and decrease in task difficulty over each block. (c) Determine the maximal duration of motor-cognitive activity, which suits the chosen intervention. This delivers the duration of the most impaired motor-cognitive activity in each block. (d) By considering total session time and number of cognitive activities, determine the number of blocks per session and the duration of the remaining activities in each block.	Supplementary S1, Section S3.4 and Supplementary Table S4
4	Variability	Define a set of variability rules for the chosen motor-cognitive exergame intervention. Preferences of the participants should also be considered.	Supplementary S1, Section S4

## 3 Results

### 3.1 Core principles

Stroke rehabilitation is driven by neuroplasticity-based (re-) learning of motor and cognitive abilities with the aim to regain daily-life functioning (55, 59). Neuroplasticity terms the experienced-based adaptation of the structure and function of the nervous system (60, 61). It has been found to be the underlying process of skill learning in animals and humans (62). Since skill (re-) learning is the key goal of stroke rehabilitation, neuroplasticity and (motor) learning principles should be considered for developing interventions for this purpose (55, 56). This may be especially important in the chronic stage after stroke, as in this phase, function regain is no longer supported by the endogenous recovery of the nervous system (63). Furthermore, general training principles should be applied in rehabilitation interventions just as for any training intervention (49, 57, 64). Precisely, objective treatment protocols based on well-reasoned rationales and with defined goals are important for a successful rehabilitation (65). To summarize, we identified three core pillars for the development of our training concept, namely principles for (1) neuroplasticity, (2) (motor) skill learning, and (3) training (Table 2).

**Table 2:** Summary of the neuroplasticity, motor learning, and training principles, and their implementation in the PEMOCS concept.

Component	Neuroplasticity principles (55)	Motor learning principles (56)	Training principles (49, 57)	Implementation in the PEMOCS concept
0) Rationale	Use it or lose it Use it and improve it Time matters Age matters		Reversibility	
1) Training Type	Specificity Repetition matters Intensity matters Transference matters Interference matters	Task-specific practice Goal-oriented practice Repetitive practice Multisensory stimulation Explicit Feedback (KR) Implicit Feedback (KP)	Exercise Type Specificity Intensity	Purpose-fully designed, user-centred, and step-based motor-cognitive exergames
2) Training dosage		Dosage Spaced practice	Volume Intervention Duration Frequency Density Time/Session duration	≥ 720 min over ≥12 weeks, 2–3 x/week on non-consecutive days, for ≥30 min
3) Progression Rules	Salience matters	Increasing difficulty	Progression Overload	Rules based on extended Taxonomy for Motor Learning (Gentile) and Performance
4) Variability rules		Variable practice (Random practice)	Variation	Rules based on extended Taxonomy for Motor Learning (Gentile) and Preference
5) Application			Periodization & programming	See Discussion

KR, knowledge of results; KP, knowledge or performance.

Combining these core principles determined the rationale and four “Components” of the PEMOCS concept (Table 2). Building the rationale, the principles “Use it or lose it”, “Use it and improve it”, “Reversibility”, and several more jointly claim that it is crucial for any individual to maintain training for sustaining and/or even improving functioning (49, 55). In line with this, it has repeatedly been shown that chronic stroke survivors can improve functioning with appropriate training interventions (66, 67). To do so, these interventions need to be well reasoned, and personally tailored (50, 51). This led us to the aim of developing a concept that bases on a standard set of rules, which can be applied in a personalized way. Building on this, we elaborated that for applying motor-cognitive training to improve cognitive functions and gait in chronic stroke, four questions (“Components”) need to be addressed; 1) What type of motor-cognitive training is used? 2) At what dosage is the training applied? 3) How is it progressed, and 4) How is it varied to achieve a standard yet personalized intervention schedule?

### 3.2 Parent theories

The Taxonomy for Motor Learning by Gentile (68, 69) was identified as a suitable framework to build the fundament of the PEMOCS concept, and was therefore chosen to be the main parent theory of our Theory Derivation process. Gentile's Taxonomy for Motor Learning is a classification system of different steps in learning motor tasks, and it explicitly considers motor learning principles. It compromises of a two-dimensional structure enabling the classification of motor tasks into different motor skill categories, which represent these different steps of learning (70). The original taxonomy has previously been used in stroke rehabilitation and guided the development of a set of exergames to promote walking ability in chronic stroke survivors (71).

In the Model of Skill Acquisition, three stages of motor skill learning were proposed (72). These stages are (1) an early or cognitive phase, where the trainee first needs to cognitively understand the task they should learn. The goal of the task and possible sub-tasks as well as their sequence are established, involving explicit knowledge. (2) An intermediate or associative phase, where the trainee starts to become more efficient in executing the task, exploring the sub-tasks and improving smoothness and coordination. Finally, (3) a late or autonomous phase, where the trainee becomes proficient in executing the task in varying versions (e.g., different speeds) and can perform it automatically (72–74). The same stages have also been described for cognitive skill learning (75), and find application in Gentile's Taxonomy for Motor Learning (68).

The Cognitive Load Theory states that learning is a process involving a limited amount of working memory, which is needed to process new information, and store it in a comparatively unlimited amount of long-term memory (76, 77). Cognitive load, the extent to which available working memory is demanded by the presented task, is recognised as a determining factor of whether learning is successful or not (77). Therefore, the Cognitive Load Theory suggests that cognitive load should be considered and measured in interventions tailoring (re)learning of tasks.

The Challenge Point Framework states that there exists an optimal level of task difficulty for promoting (motor) learning, and that this optimal level relies on the skill level of the learner (78). Applying that, Guadagnoli et al. suggest to divide task difficulty into nominal, which describes the objective difficulty of a task regardless of the performers skill level or the conditions the task is performed under; and functional, which considers the performer's skill level and the prevailing conditions (78). The Challenge Point Framework claims that, when planning rehabilitation interventions, it is important to consider functional task difficulty instead of only nominal task difficulty (78). This means that rehabilitation interventions must be personalized, which aligns well with recent research guidelines for rehabilitation [(48, 49), see also “1.1 Component 1: Progression Rules”].

The Flow Theory describes a state of “optimal experience”, which explains the motivation and commitment of human beings to activities without obvious external rewards (79). Nine characteristics are proposed to be key attributes of the flow state; challenge-skill balance, action-awareness merging, clear goals, unambiguous feedback, concentration on task at hand, sense of control, loss of self-consciousness, transformation of time, and autotelic experience (79). These can be applied to games including video games, for example in so-called Serious Games or Exergames, which has been described in the GameFlow model (80). The GameFlow model links design of different games to enjoyment of the activity and presents criteria, which can distinguish enjoyable and less enjoyable games (80). Moreover, flow is increasingly measured when investigating neurorehabilitative interventions, as it has been remarked that flow can be beneficial for the success of therapy (81).

The Guided Plasticity Facilitation Model explains how physical and cognitive activities may interact in neuroplasticity. It suggests that physical activity of sufficient intensity facilitates plasticity, while concurrent cognitive activity guides the neuroplastic changes (82, 83). The model claims that both, physical and cognitive activity, are necessary for the induction and retention of neuroplastic changes. Therefore, combined motor-cognitive interventions may specifically trigger neuroplasticity (84). As one of our core principles states that neuroplasticity is central to target in chronic stroke, this corresponds well with our aim to use motor-cognitive exergame interventions in the PEMOCS concept.

FITT-VP stands for the training principles Frequency, Intensity, Time, Type, Volume, and Progression (85, 86). The FITT-VP principles should be applied in any training intervention for any trainee, therefore, also for rehabilitation interventions. The application of the FITT-VP principles also helps to personalize the interventions (85, 86).

The Theoretical Model to Describe Progressions and Regressions for Exercise Rehabilitation describes how a physiotherapy intervention should be structured to achieve successful regain of the target function (87). It claims that the core task of the target function should first be introduced in the simplest version, and then progressed by manipulating internal variables such as speed or range of motion. As soon as the patient is proficient in executing this simple version, an extrinsic component can be added such as a distracting environment or an external force applied. This will lead to a performance reduction compared to the earlier condition, for example in terms of acuity or speed. The task should then be trained under the new condition until proficiency is reached again, before adding a new extrinsic component. By this step-wise introduction of extrinsic components of increasing difficulty, the patient is guided to regain the target function under varying conditions in a standardised yet flexible way (87). This model of progression corresponds well with Gentile's Taxonomy for Motor Learning and the Model for Skill Acquisition described above.

### 3.3 What to keep: adoptions from the main parent theory

**Figure 1:**
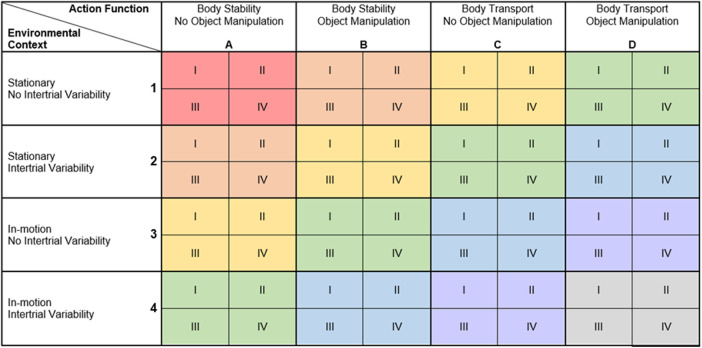
Extended taxonomy based on Gentile's taxonomy for motor learning (88). (**A–D**) sub-dimensions of action function (body-intern task levels); 1–4; sub-dimensions of environmental context (body-extern task levels). I–IV; cognitive dimension, domains are arranged based on baseline assessments (see 3.3.1); I, least impaired; II. second least impaired; III: second most impaired; IV, most impaired. Colours, Difficulty levels based on (71); red, level 1; orange, level 2; yellow, level 3; green, level 4; blue, level 5; purple, level 6; grey, level 7.

Gentile's Taxonomy for Motor Learning (70) was chosen as fundament of the PEMOCS concept and to build its basic structure. It comprises of a table with two dimensions with two sub-dimensions each (2 × 2 structure, Figure 1). The dimension “Action function” on the *x*-axis represents the body-internal level of a task and is sub-divided into the dimensions “Body Stability vs. Body Transport”, and “No Object Manipulation vs. Object Manipulation”. “Environmental context” on the *y*-axis represents the body-external level of a task, and includes the sub-dimensions “Stationary vs. In-motion”, and “No Inter-trial Variability vs. Inter-trial Variability” (70). Combining all sub-dimensions results in 16 motor-skill categories (Figure 1). Task difficulty increases from category 1A at the top left to 4D at the bottom right, which enables step-wise progression through the classification system (Figure 1, (71)). By that, Gentile's Taxonomy for Motor Learning builds a standard framework, through which a learner can move in a personalized manner. The increase in task difficulty by step-wise introduction and then combination of the (sub-) dimensions suits the progression steps suggested in Blanchard's Progression Model for Exercise Rehabilitation (87). It also aligns with the three stages of motor and cognitive skill learning in the Model of Skill Acquisition [see section 3.4.3.4 (72, 75)]. Moreover, besides providing overload by increasing task difficulty, Gentile's Taxonomy for Motor Learning may also provide progression in terms of cardiovascular intensity (55, 57), as introducing “Body Transport”, “Object Manipulation”, “Inter-trial Variability”, and “In-motion” may produce higher cardiovascular load compared to the easier sub-dimensions. This 2 × 2 structure of Gentile's Taxonomy for Motor Learning was adopted for the PEMOCS concept, as it builds a standardised scheme of increasing task difficulty and allows for variability of exercises, which can be applied to individual participants in a personalized manner.

### 3.4 What to add and change: the PEMOCS concept

Before starting with the modifications on Gentile's Taxonomy for Motor Learning, we remarked at this point that the components “(1) Training Type” and “(2) Training Dosage” (Table 2) needed to be added to the training concept first. Therefore, the following two sections summarize our findings of the narrative review regarding the questions, “*how to choose a type of exergame training*?”, and “*at what dosage to apply it*?”. The further development of Gentile's Taxonomy of Motor Learning, modifications, and further additions were based on these findings.

#### 3.4.1 Choosing the type of motor-cognitive exergame training

Many exergame-based rehabilitation interventions are performed more readily and more likely repeated compared to conventional therapy interventions (89, 90), which can largely be explained by the high levels of reported enjoyment using such programs (91). This is important as a high number of repetitions is key for both, triggering neuroplasticity and promoting skill learning (55, 56). Motivating a patient to repeat an exercise multiple times is often difficult, as boredom and exhaustion may hinder them. This can lead to low adherence to rehabilitation interventions and, therefore, limit their success (92). Exergames make use of two powerful tools for increasing motivation in rehabilitation; (non-immersive) virtual reality (VR) and gamification (93, 94). Therefore, the high motivational power of exergames, which can be deduced from the GameFlow model (80) and a recent meta-analysis (31), is highly beneficial.

Besides being very motivating, the exergame training used to implement the PEMOCS concept should be user-centred and purposefully designed to meet all the requirements for training to enhance (motor) skill learning [Table 2, (56, 95)]. (1) Such purposefully-designed and user-centred exergames provide clear goals, which need to be achieved (“Goal-oriented practice”). (2) They provide frequent and immediate explicit and implicit feedback addressing different sensory modalities similarly to daily life (96, 97), which fulfils the principles “Multisensory stimulation”, “Knowledge of Result”, and “Knowledge of Performance”. (3) Purposefully-designed and user-centred exergames provide the opportunity to continuously adapt stimuli allowing variable practice and progression (41, 98). (4) The use of (non-immersive) VR further helps the trainee to direct their attention external—on the target instead of their movement -, which has been shown to enhance motor learning (99). All of this directly aligns with the characteristics of the flow state [Flow Theory (79)], which again promotes enjoyment of the activity, and improves adherence (91).

Moreover, motor-cognitive exergames may positively influence the neuroplasticity principles of “Transference” and “Interference” [Table 2, (55)]. Both can occur when a training induces neuronal plasticity, however, transfer effects to other functions are generally welcome in rehabilitation settings (e.g., a specific training leads to better daily-life performance), while interference effects should be minimized (e.g., two therapeutical interventions interfere and, thereby, hinder each other's effects) (55). Motor-cognitive exergames, in contrast to single cognitive trainings, have been shown to produce transfer effects to untrained cognitive functions (100). A possible mechanism of this success may be that by exergaming, the trainee learns/trains how to learn, which then improves coping with new tasks in daily life (100). Furthermore, integrated motor-cognitive tasks such as exergames may avoid interference effects as the motor and cognitive sub-tasks share one goal, which is why none of them has to be prioritized [such as in a classical dual-task, (19)]. Therefore, and as the motor and cognitive systems are intertwined (16, 17), integrated challenges may overcome the hurdle of interference and even lead to enhanced neuroplasticity in both systems [compare “guided plasticity facilitation” model (82, 83, 101)].

To further be specific in targeting gait (neuroplasticity/motor learning/training principle of specificity), exergame training integrating functional stepping movements may be most beneficial (102–104). This matches well with recent findings, that also for improving cognitive functions, exercise in a standing position focusing on step-based movements may be most beneficial (105, 106). Therefore, we suggest using motor-cognitive exergame trainings, which are user-centred, purposefully designed to meet neuroplasticity and motor learning principles, and integrating stepping movements in a standing position to implement the PEMOCS concept in chronic stroke patients.

#### 3.4.2 Defining the training dosage

The dosage of a training intervention can be defined using exercise variables derived from the FITT-VP principles or the duration of the intervention (49, 57). Aiming to apply these variables, we found the following evidence in literature.

The recommended minimum intervention duration of a motor-cognitive intervention to induce motor and cognitive improvements has been investigated by several systematic reviews, and it seems that adaption in motor functions requires less time than adaptation in cognitive functions [e.g., (97, 107), more references see below]. An intervention duration of at least eight weeks has been recommended for VR interventions and walking training to improve motor functions in stroke survivors (97, 108). Confirming these recommendations, VR and motor-cognitive interventions lasting eight weeks or even less were found to superiorly improve walking and balance in stroke patients compared to active and passive control groups (26, 28, 109). To improve cognitive functions in stroke patients and older adults, however, interventions should last at least twelve weeks (84, 107, 110, 111). This may be the reason why a systematic review on the effect of VR training in stroke patients found no superior effect of VR over active or passive control groups on cognitive performance (112). In this review, the study interventions had a maximum duration of eight weeks, which may have been too short for cognitive adaptions in the stroke patients. Two other reviews regarding neurological and general populations, on contrast, found superior effects on cognitive functions of exergaming compared to active or passive control groups (41, 42). These two reviews reported average interventions durations of ten weeks, and both included studies with interventions lasting up to 24 weeks.

Recommendations regarding frequency and time (session duration) also differ for motor and cognitive functions. It seems that for improving motor functions, shorter sessions with high weekly frequency are beneficial, while cognitive functions benefit more from less sessions per week of a longer session duration each (33, 108, 111). A meta-regression on gait training and a systematic review on exercise in stroke jointly recommend at least three sessions per week of 30 min or more for improving motor functions and specifically gait ability (108, 113). For improving cognitive functions, however, 2–3 exercise sessions per week of 45–60 min were found most effective (111, 114). What was common for both, motor and cognitive functions, is that a daily frequency (≥4 session per week) may reduce the effectiveness of the intervention (97, 107). This underpins the principles “Density” and “Spaced practice”, which consider the necessity of rest periods, e.g., training-free days between sessions, to enable neuronal adaption (49, 56). Therefore, the recommended 2–3 sessions per week should be spread over the week, enabling “Spaced practice”. Systematic reviews, which found significant superior effects of motor-cognitive and VR interventions compared with active or passive control groups on motor and cognitive functions in stroke and neurological populations, reported sessions of 20–60 min, two to five times per week (26, 31, 41, 42, 96).

The physical exercise intensity in stroke rehabilitation intervention should generally be at least moderate, on the one hand to induce neuroplasticity via the release of neurotrophic factors [which appears to require at least moderate exercise intensity (115, 116)], and on the other hand for influencing cardiovascular risk factors (117). Guidelines for exercise intensity state that moderate or higher intensity exercise should include activities of at least 60% HRpeak or rated with at least 12/20 on the traditional Borg scale for exertion (118). High-intensity exercise has been found more beneficial than moderate to low intensity exercise for improving walking competency in stroke survivors (113). For improving cognitive functions in stroke survivors, low to high intensity exercise has been found beneficial (119–121). Confirming that, motor-cognitive trainings at moderate physical intensity have been found to improve cognitive functions and gait in healthy older adults (107, 122). Therefore, motor-cognitive exergame training achieving at least moderate physical intensity seems to be most suitable for the PEMOCS concept.

Finally, the overall volume of an intervention is an important training principle. To induce changes in motor and cognitive functions by VR and motor-cognitive interventions, several reviews considering stroke patients and healthy older adults recommend 900 or 720–1,000 min of overall training time, respectively (84, 107, 123). Again, motor outcomes seem to take less time for improvement. In three systematic reviews, balance and gait outcomes improve superiorly compared to active and passive control groups with average intervention durations below 720 min (28, 33, 124). While cognitive outcomes were found to improve more in systematic reviews where the exergame interventions lasted on average longer than 1,000 min (41, 42). An exception is the review by Aminov et al. who found that VR interventions compared to active or passive conventional rehabilitation significantly more improved cognitive functions at an average intervention duration of 685 min (125).

Based on these recommendations, we concluded that a motor-cognitive training for improving cognitive functions and gait in chronic stroke survivors should include at least 720 min of moderately to highly intensive training over a period of twelve weeks or more, in 2–3 sessions on non-consecutive days a week, which last approximately 45 min.

#### 3.4.3 Progression rules

After having collected evidence on different types of motor-cognitive exergame interventions, and at what dosage they should be performed, we returned to the fundament of the PEMOCS concept, the personalized progression of the training tasks, which bases on Gentile's Taxonomy for Motor Learning (68). To apply it to motor*-cognitive* exergame training, we extended the framework by a third, cognitive dimension. To do so, we sub-divided each of the 16 motor-skill categories into cognitive sub-dimensions, which represent different cognitive domains. These cognitive domains should focus on cognitive deficits identified in community-dwelling chronic stroke patients (126, 127). We present the PEMOCS concept with four cognitive sub-dimensions (I–IV, Figure 1) in this paper. Each motor-skill category is sub-divided into four cognitive sub-dimensions, resulting in 64 motor-cognitive skill categories (Figure 1). However, if it is practically compatible with the other rules of the training concept, the number of cognitive sub-dimensions can be any (e.g., if an intervention targets six cognitive domains, there would also be six cognitive sub-dimensions, resulting in 96 motor-cognitive skill categories). This extended taxonomy builds the foundation of the PEMOCS progression rules, and includes the two key innovations of the training concept: the (1) standardised rules for personalisation of progression of (2) not only motor, but motor-cognitive exergame training. Further extensions were based on the other parent theories and are presented in the following.

##### 3.4.3.1 Personalizing the allocation of the cognitive sub-dimensions

The first step of personalization in the PEMOCS concept is enabling a focus on the most impaired cognitive function(s) for each individual participant. To do so, the cognitive sub-dimensions are ordered from “least impaired” to “most impaired” for each individual participant. This is done using suitable neuropsychological assessments, which are performed before the start of the intervention. For an example how to use assessments to rank the cognitive domains, see our application example (Supplementary S1, Section S3.1.2). Having determined the order of the cognitive sub-dimensions for a specific participant, they are then arranged in the extended taxonomy. Following the overall structure of the taxonomy with “easiest” in the top-left and “most difficult” in the bottom-right of the table, the least impaired domain of a patient is placed in the top-left square (I), and the most impaired domain in the bottom-right square (IV) of each motor-skill category (Figure 1, example with four cognitive domains).

##### 3.4.3.2 Assigning motor-cognitive tasks to the sub-dimensions of the extended taxonomy

All activities of a motor-cognitive exergame training (e.g., different games and game versions such as in the application example, Supplementary S1) now need to be assigned to one of the motor-cognitive skill categories of the extended taxonomy. To do so, the definitions of the sub-dimensions (68, 69, 71) should be used as follows:

• “Stationary” vs. “In-motion”: Activities that take place within stationary surroundings should be assigned to rows 1 and 2 of the dimension “Environmental context”. This includes activities where tasks are executed in still game scenes. Activities taking place in moving game scenes, should be placed in the rows 3 and 4.
• “No Inter-trial Variability” vs. “Inter-trial Variability”: Exercise sets that comprise of a series of the exact same task, which is repeated again and again under the same conditions, should be assigned to rows 1 and 3 of the dimension “Environmental context”. Exercise sets, however, during which the task is varied, e.g., in terms of inter-stimulus interval or direction, should be assigned to rows 2 and 4.
• “Body Stability” vs. “Body Transport”: Activities that require a stable body position, e.g., balance exercises with which the trainee is required to maintain a stable position of its avatar in the game, are assigned to columns A and B of the dimension “Action function”. If the body is required to move for accomplishing the goals in the game, however, the activities are assigned to columns C and D.
• “No Object Manipulation” vs. “Object Manipulation”: Tasks that include no object manipulation in the virtual environment, e.g., simple reactions to appearing stimuli, should be placed into columns A and C of the dimension “Action function”. Tasks, on the other hand, which require the manipulation of an object in the virtual environment, e.g., moving an avatar to collect points or avoid obstacles, are placed into columns B and D.
• Cognitive Dimension: The main cognitive target domain of each purpose-developed exergame needs to be identified. Based on this categorization, the exergame activities are assigned to the cognitive sub-dimensions (I–IV in our example with four cognitive domains). We recommend that the categorization is be made by an experienced neuropsychologists to ensure the content validity of the exergames used to train each cognitive domain.

The goal should be to fill as many of the motor-cognitive skill categories with different motor-cognitive tasks and variations thereof to enable the application of the progression and variability rules described in the next sections.

##### 3.4.3.3 Progression from session to session

Now that the core structure of the progression rules was determined using the extended taxonomy (Figure 1), it needed to be defined when and for how long the motor-cognitive activities allocated to different motor-cognitive skill categories should be performed by the trainees. To do so, the extended taxonomy was divided into seven difficulty levels as suggested by (71) (Figure 1):

• 1: red, including motor-skill category 1A
• 2: orange, including motor-skill categories 1B, 2A
• 3: yellow, including motor-skill categories 1C, 2B, 3A
• 4: green, including motor-skill categories 1D, 2C, 3B, 4A
• 5: blue, including motor-skill categories 2D, 3C, 4B
• 6: purple, including motor-skill categories 3D, 4C
• 7: grey, including motor-skill category 4D

**Figure 2:**
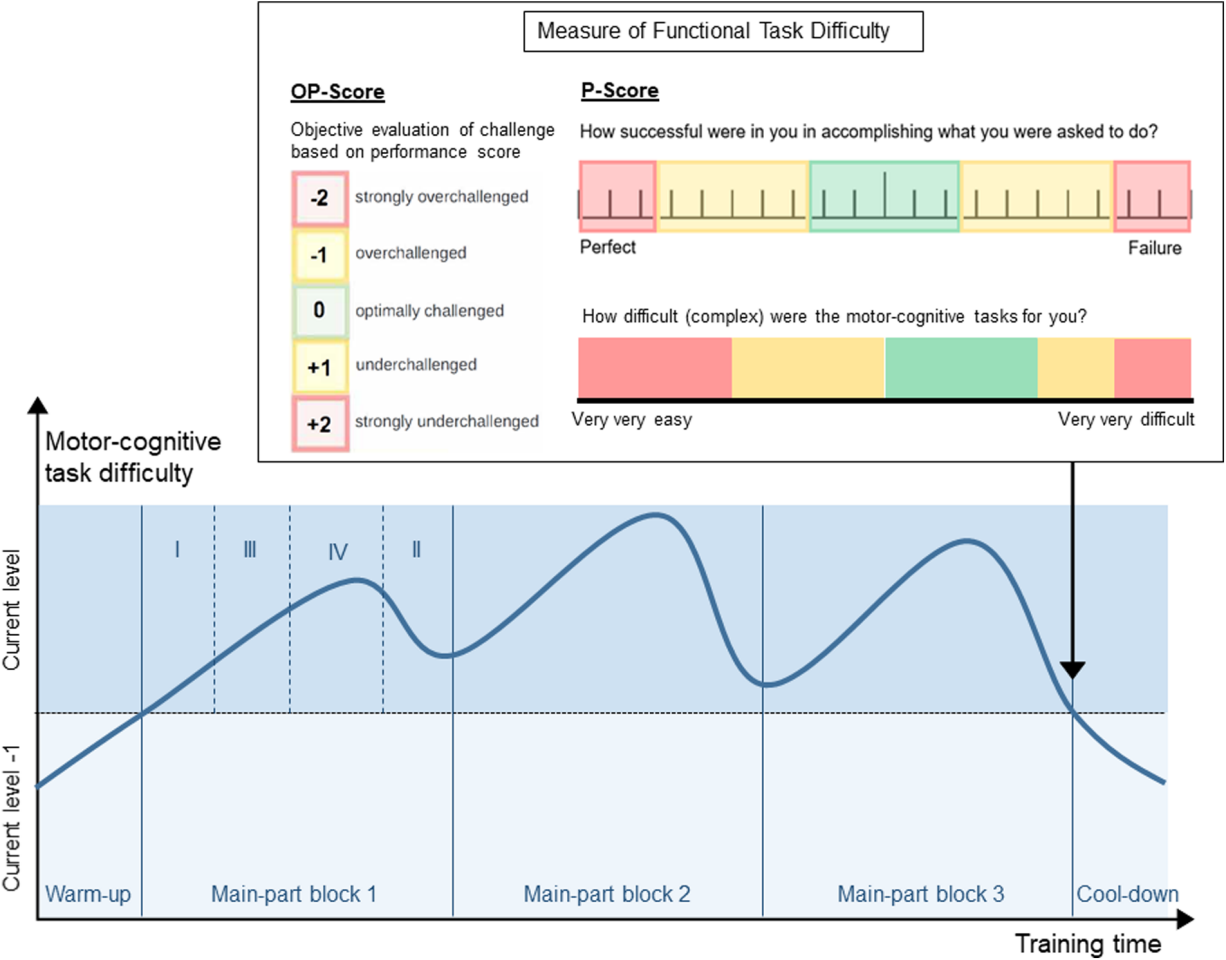
Example overview of a training session with warm-up, three blocks in the main-part, and cool-down. Each block contains four activities (as shown in block 1). To achieve the shown curve of motor-cognitive task difficulty, the four activities in each block target one of the four cognitive domains (domain I—least impaired domain; domain IV—most impaired domain). “Measure of functional task difficulty”: Supervisor's scale of objective performance (left), rating scale for perceived performance (top-right), rating scale for perceived task difficulty (bottom-right). The objective and subjective evaluation of the participant's challenge are collected after the last of block of the main-part.

Each training session includes activities from within one difficulty level. From session to session, participants individually move from difficulty level to difficulty level, back and forth, with the goal to achieve optimal task difficulty for skill learning at each time point. Personalized adaption of task difficulty leads to superior learning compared to a fixed progression (56, 128). Moreover, this provides that the tasks are at all time points matched to the participants' skills, which may promote the flow state (79). The progression process is based on the Challenge Point Framework (78). Skill learning has been found to be optimal at a moderate to high functional task difficulty (129). We suggest measuring functional task difficulty in two ways, which are based on the Cognitive Load Theory (77, 130, 131). On the one hand, an objective evaluation of how strongly the participant was challenged should be made. This objective evaluation can be based on any performance score determined during the training (e.g., provided by a technology-based training system). This objective evaluation of challenge must be able to deliver a score from −2 (strongly overchallenged) to +2 (strongly underchallenged) (OP-score, Figure 2, left of “Measure of Functional Task Difficulty”). On the other hand, the participants' subjective evaluation of their own challenge should be considered, as it has been shown that having control over the level of task difficulty improves skill acquisition and retention (132). To do so, participants rate their perceived performance (PP) and perceived task difficulty (PTD) in each session over all activities. The ratings of the perceived performance are based on the performance sub-score of the NASA-Task Load Index (133), using a visual analogue scale (VAS) labelled from «perfect» (left side) to «failure» (right side) (Figure 2, top right of “Measure of Functional Task Difficulty”). Based on the Cognitive Load Theory (131), subjective ratings of the perceived motor-cognitive task difficulty are gathered using a VAS (Figure 2, bottom right of “Measure of Functional Task Difficulty”) labelled with «very, very easy» (left side) to «very very difficult» (right side). Visual analogue scales are used as they support a more accurate rating by giving the opportunity to rate on a continuum instead of predefining specific options (135). For both scales, target areas for optimal functional task difficulty (green) as well as over- and under-challenge (yellow—red) were determined based on the optimal workload for motor learning established in (129). Based on these target areas, the subjective ratings of the participant are transferred into challenge scores from −2 (strongly overchallenged) to +2 (strongly underchallenged) and averaged (P-Score, Figure 2).

These objective and subjective measures of functional task difficulty are then combined considering the type of objective evaluation (e.g., the three scores may be averaged, see Supplementary S1, Figure S3). This delivers the progression steps, which are the amount of difficulty levels a participant progresses from one session to the next. If the participant was optimally challenged, they remain in the same difficulty level (progression steps = 0). In case they were underchallenged or even strongly underchallenged, they progress one or two level(s), respectively (progression steps =  + 1/ + 2). In case they were overchallenged or strongly overchallenged, however, they retrogress one or two level(s), respectively (progression steps = −1/−2). Following the personalized progression, the next training session will again be planned within the optimal difficulty level for the participant.

##### 3.4.3.4 Progression within sessions

Each session contains a warm-up, a main-part, and a cool-down (Figure 2). The warm-up and cool-down include activities from a difficulty level below the current level, as participants have already acquired proficiency in these tasks [autonomous phase (72)]. This provides a suitable (re-)familiarisation with the training, and a high feeling of fun and success, respectively. The main-part is performed with tasks from the current difficulty level, where participants are in the associative and cognitive phase (72). It is divided into at least two training blocks of individual length (Figure 2) separated by short breaks (59). Each block contains as many activities as cognitive sub-dimensions have been defined in section 3.4.3.1 (for example four, Figure 2). Task difficulty should increase, peak, and decrease again in each block as well as over the whole session (line in Figure 2). So, in our example with four cognitive domains, the first activity targets the least impaired domain (I), the second activity targets the second most impaired domain (III), the third targets the most impaired domain (IV), and the last activity targets the second least impaired domain (II). To set a focus on the most impaired domain, the third activity in each block lasts longer than the other activities (Figure 2). The most difficult motor-cognitive activities are performed in the second block. This curve of task difficulty provides a learning-supporting alternation of tasks, where participants are in the associative and cognitive learning phases (59, 74). To determine the number of blocks, and the duration of each activity in these blocks, a maximal duration of the activities is first defined, most probably on practical reasons (e.g., how long can a patient perform an activity, what makes sense to prevent boredom). This equals the duration of the activity in the most impaired domain in each block, which should receive maximal attention. Additionally, the number of cognitive domains (determined in D.3) determines the number of activities per block. From this, the most appropriate number of blocks per session and the duration of each single activity should be determined.

At the end of the main-part, the objective evaluation and subjective ratings of perceived motor-cognitive task difficulty and perceived performance are collected to prevent that participants consider the easier cool-down activity for their ratings [Figure 2, (135)].

#### 3.4.4 Variability rules

By moving through the extended taxonomy (Figure 1), participants experience variability by being confronted with new motor-cognitive tasks in every difficulty level they progress to. By applying the progression within each session, participants are confronted with tasks targeting all cognitive domains, which provides variability within each session. Moreover, variability can be kept up by performing all possible motor-cognitive tasks at least once a week, and thereby considering the preferences of the participants. Depending on the chosen motor-cognitive exergame, additional rules can be defined to ensure variability throughout the training intervention.

### 3.5 Refining steps after feasibility study

In the feasibility study being part of the developmental process of the PEMOCS concept, a first draft of the concept with the same motor-cognitive exergame training as described in the application example (Supplementary S1) was implemented and evaluated. This draft was described in (54), where we also identified three main weaknesses of the concept. Following up on this, we describe here what was refined after the feasibility study to address these weaknesses and improve the concept. A more detailed description of the three weaknesses can be found in “Secondary Outcomes—Strengths, and Limitations of the Adapted Taxonomy” in (54).

#### 3.5.1 Motor and cognitive progression should be coupled

In the draft, separate ratings for motor and cognitive task difficulty were collected to guide the personalized progression. This led to application problems in patients with unequal motor and cognitive deficits, and moreover, did not take into account how intertwined motor and cognitive skill learning is [see Model of Skill Acquisition (72, 75)]. We, therefore, decided that motor-cognitive challenge instead of separate motor and cognitive task difficulty should be inquired from the participants to perform the personalized progression. We identified the difficulty levels presented in (71) as suitable tool to progress coupled motor-cognitive task difficulty, and established the progression steps, which would guide the pro- and retrogression between the levels.

#### 3.5.2 More objective assessment of the participants' challenge

In the draft, the only measure that guided the progression were subjective ratings of task difficulty provided by the participants at the very end of the session. This resulted in motor-cognitive challenges below the targeted range (54). We discussed that subjective task-difficulty ratings alone are inappropriate to guide the motor-cognitive progression (54). To improve this, we did twofold. On the one hand, we added the OP-score to the progression rules (see section 3.4.3.3), which includes an objective measure of the participants' challenge into the progression rules. Unfortunately, it was not possible to elaborate a concrete recommendation for this objective parameter in the meantime (see Future Directions and Limitation). For the application example, we integrated the supervisor's evaluation of the participant's challenge as a first approach towards more objective progression steps (see Supplementary S1, Section S3.3). On the other hand, we extended the subjective estimate of the participants’ challenge by a second rating, the perceived performance. Perceived performance has been shown, along with perceived task difficulty, to help discovering the functional task difficulty for a participant (129). Besides this, we redefined that participants rate their perceived task difficulty and perceived performance now during the main part or directly after it, instead of at the end of the session, when the cool-down game may be most present in the participants’ minds.

#### 3.5.3 Fill empty motor-cognitive skill categories

In the draft, we neglected two sub-dimensions (one in each dimension) to first get an insight into how the structure could be implemented practically. This implied that a different set of rules defined how the motor-cognitive tasks were assigned to the skill categories (54), and many categories remained empty. We redefined these rules (see “D.3.2) Assigning Motor-Cognitive Activities to the Sub-Dimensions of the Extended Taxonomy”), coming closer to the original definitions of the sub-dimensions in Gentile's Taxonomy for Motor Learning (68). In any application of the PEMOCS concept, it should be strived to fill all motor-cognitive skill categories with at least one activity. This can be achieved by creating new exergames, adapted exergame versions of the original exergames that fit other sub-dimensions, and adding secondary tasks. Applying this, we (1) developed two completely new exergames in the refining process (Shopping Tour and Gears, see Supplementary S2); (2) adapted existing exergames to other motor-cognitive skill categories (e.g., Nomis, which the task of the original game Simon is reversed to target another sub-domain of memory functions, Supplementary S2); and (3) integrated a variety of motor and cognitive secondary tasks to increase the task difficulty and intensity of the training (e.g., dribbling—jogging on the spot while playing the game, see Supplementary S1, S2).

## 4 Discussion and conclusion

This methodological paper describes the rationale behind the development of a standardized concept for personalized motor-cognitive exergame training to improve cognitive functioning and gait in community-dwelling chronic stroke survivors. After the (sub-)acute phase, meaning from six months post-stroke, stroke survivors nowadays receive no or insufficiently intense care (3, 136). This can lead to impaired daily-life functioning and reduced health-related quality of life (3). Therefore, future health care needs solutions for providing more and sufficiently intense training to chronic patients (7). Motor-cognitive exergame trainings, including self-reliant and home-based trainings, have been recommended to address this need (31, 42, 137). However, exergame training has so far mainly been applied in an unstructured manner, which may limit their effectiveness (28). We, therefore, developed an evidence-based training concept considering neuroplasticity, motor learning, and training principles, to guide long-term and personalized exergame training application in chronic stroke survivors. For this purpose, we followed the steps for a Theory Derivation procedure (52) including literature research, identification of parent theories, determination of what to keep from the parent theory, and integrations of additions and modifications. Additionally, we considered some aspects of the “Framework for Developing and Evaluating Complex Intervention” by the Medical Research Council (53). We identified a suitable intervention, developed a first draft of the training concept and tested its feasibility, refined the concept, and will now implement it (53).

We identified Gentile's Taxonomy for Motor Learning (68) as suitable fundament for the training concept, and extended by a third cognitive dimension for the implementation with motor-cognitive training. Gentile's taxonomy provides a standard categorization of tasks according to their nominal task difficulty, which can be personally applied for each participant ensuring that each can train at their individual optimal functional task difficulty (78). We defined rules how this personal application should be progressed and varied, which were inspired by further models and concepts from related research and therapy fields. The resulting PEMOCS concept can be applied to any motor-cognitive exergame intervention, which is performed with the aim to improve cognitive functions and gait. It was developed with a focus on community-dwelling chronic stroke survivors; however, as it was also based on literature covering general neurorehabilitation and training, it may have the potential to guide the application of motor-cognitive exergame interventions in other populations, such as healthy older adults or other neurological patients.

### 4.1 Suggestions on how to apply the PEMOCS concept

The PEMOCS concept is intended to serve as a guide for structuring and implementing motor-cognitive training in the rehabilitation of (chronic) stroke. It can be applied in different settings (e.g., inpatient or outpatient rehabilitation, physical therapy, secondary prevention, or similar) and with different equipment (e.g., different VR or exergame systems). As described above, we suggest using a user-centred, purpose-fully designed, and step-based motor-cognitive exergame training to apply it (84, 90, 138). To implement the progression and variability rules based on Gentile's Taxonomy for Motor Learning, an ideal way would be to design a motor-cognitive exergame intervention fitting the different dimensions and sub-dimensions. For example, novel exergames may be designed such as in (71), where six new games were developed specifically meeting the requirements of Gentile's Taxonomy for Motor Learning. The new games require the player to perform balance and stepping tasks with the aim to improve walking in stroke survivors (71). However, it is also possible to assign existing activities to the motor-cognitive skill categories, as we suggest in the “Application example” (Supplementary S1) and (54). To determine the appropriate dosage of the intervention, the above-described recommendations and further practical implications should be considered. For example, the access to the training may play a role, or it should be contemplated if participants need supervision (which would limit the dosage more than if participants can perform the training self-reliantly).

Depending on the setting, the PEMOCS concept may be used to structure a single intervention period or to guide continuous motor-cognitive training in chronic stroke survivors. In in- or outpatient rehabilitation with limited duration, an application similar to the way presented in the application example may be appropriate (Supplementary S1). Such a training period of 12–16 weeks may also be repeated e.g., once or twice a year, if practical reasons (such as patients' schedules) and limited costs [e.g., therapy time, which is paid by the health insurance, (139)] make it more applicable than continuous training. This would consider the training principle of periodization [Table 2, (49)]. Blocked periodization is applied in all types of training schedules, for instance in professional athletes, and was found beneficial as it seems to boost adaption and residual training effects (140). It would also be similar to forced use protocols, such as constraint-induced movement therapy, where intensive training over days or weeks is followed by less intense or resting periods (141). Forced use protocols are effective in improving functioning after stroke (142, 143). Nevertheless, continuously maintaining exercise with the goal to regain functioning and for secondary prevention may be more beneficial for chronic stroke patients (144). For aiding this, exergame training following the PEMOCS concept can be a complement to usual care or help prevent deconditioning when therapy is discontinued (42). Chronic stroke survivors can be guided to maintain a continuous motor-cognitive exercise regimen making use of the progression and variability rules presented here. As long-term continuous face-to-face care is usually not covered by health-care systems (139), an application in a telerehabilitation setting may be more appropriate than the supervised way presented in our application example (Supplementary S1). A telerehabilitative application would mean that patients train self-reliantly at home using technology-based training systems such as exergames, while standing in regular remote contact with an instructing health-care professional (145). For this, the identification of a reliable objective performance parameter (see OP-score, section 3.4.3.3) would be necessary (see Future Directions and Limitations). Moreover, as the long-term use of the same exergames can lead to boredom, we recommend the consideration of further instruments for variability for this application, for instance unlocking new games, adding new levels or changing the exergame system after some time (146).

## 5 Future directions and limitations

The PEMOCS training concept should now be implemented in a clinical study to evaluate its effects on cognitive functions and gait in chronic stroke survivors. To do so, a randomized controlled trial (RCT) is currently conducted [(58), clinicaltrials.gov, NCT05524727] using the application example in Supplementary S1. Besides cognitive functions and gait, mobility under single- and dual-task conditions as well as health-related quality of life will be secondary outcomes of this study. A limitation in the developmental process of the PEMOCS concept was that no focus group study or other integration of clinical experts was performed. A second limitation of the PEMOCS concept as presented here is that it was not possible to suggest a concrete objective measure of the participant's challenge. There may be exergaming systems that provide performance parameters, which enable the identification of a threshold at which participants should progress or retrogress to another level. However, for other systems, which do not provide such parameters (as it was the case for the exergame system used in the application example, Supplementary S1), it would be desirable to have other objective ways for measuring the participant's challenge. Future research may investigate physiological parameters such as heart rate variability, breathing patterns, body temperature, or skin conductance for this purpose (138, 147, 148), which may have the advantage of being independent of a specific training type or system. We considered this gap of knowledge when developing the PEMOCS concept, and the integration of a truly objective parameter to determine the progression steps will be unproblematic.

## Data Availability

The original contributions presented in the study are included in the article/**Supplementary Material**, further inquiries can be directed to the corresponding author.
