# Supplementary material for: PEMOCS: theory derivation of a concept for PErsonalized MOtor-Cognitive exergame training in chronic Stroke—a methodological paper with an application example
Source: Front Sports Act Living. 2024 Jun 10;6:1397949. doi: 10.3389/fspor.2024.1397949 (PMC11194322; doi:10.3389/fspor.2024.1397949)
Supplement: Supplementary file 1 [file Datasheet1.pdf]

Supplement 1: Application example of the PEMOCS concept

S.1) Training Type

An appropriate tool to apply the PEMOCS concept, is the step-based, exergaming system "Dividat Senso" (Dividat AG, Schindellegi, Switzerland), which meets all prerequisites we described in 'D.1) Choosing the Type of Motor-cognitive Training'. The "Dividat Senso" is feasible to use in older adults and neurological patients (Swanenburg et al. 2018, Swanenburg et al. 2020, Altorfer et al. 2021, Huber et al. 2021, Schättin et al. 2021, Seinsche et al. 2022, Manser et al. 2023). First studies showed positive effects of the exergame training with the "Dividat Senso" in neurological and geriatric patients (Altorfer et al. 2021, Swinnen et al. 2021, Jaggi et al. 2023), while further projects are currently running (Manser et al. 2023, Seinsche et al. 2023). The "Dividat Senso" provides cognitively challenging video games, which are presented on a screen in front of the participant, who stands on a pressure-sensitive plate. The participant interacts with the video games by performing whole-body movements such as body-weight shifts, steps, and squats on the plate (compare motor tasks in Table S4). For a detailed description of the "Dividat Senso", see (Huber et al. 2021). In this application example, participants complete all training sessions supervised by a trained study-staff member (training supervisor).

S.2) Training Dosage

Based on the recommendations described in 'D.2) Defining the Training Dosage', the PEMOCS concept will be applied with the following training principles (Table S1); in the planned RCT, participants will train twice a week, 30-40 minutes over 12 weeks, resulting in a total training volume of 840 minutes. The following practical aspects played a role. Twelve weeks was assumed a feasible duration for the study intervention, especially when striving for an acceptable recruitment rate. Regarding frequency, we found in our feasibility study that two sessions per week were feasible for an on-site training intervention at rehabilitation centres, sessions of 30-40 minutes were most accepted (Huber et al. 2021). Time progression will be applied by adding two minutes of training every week (Table S1).

**Time point**	**Frequency**		**Time** [min]		**Volume / Week** [min]
week 1-2	2	x	30	=	60
week 3-4	2	x	32	=	64
week 5-6	2	x	34	=	68
week 7-8	2	x	36	=	72
week 9-10	2	x	38	=	76
week 11-12	2	x	40	=	80
**Total Volume** [min]				=	**840**

Supplementary Table S1*: Training variables of the application example of the PEMOCS concept.*

S.3) Progression Rules

The "Dividat Senso" provides a selection of games, which target different cognitive functions. Most games train several functions, however, for all of them a main target function can be defined, which can be categorized into attention (AF), working memory (WM), executive functions (EF), memory (MF), and visuospatial functions (VSF) (for a description of all games, see Supplement 2). AF and WM were fused into one cognitive sub-dimension out of two reasons, (1) the task measuring working memory in the MoCA is counted in the domain of AF (Nasreddine et al. 2005), which plays a role as the MoCA is used to allocate the domains to the extended taxonomy (see below, section S.3.1). (2) Allocating the working memory game to the AF sub-dimensions helped filling the motor-cognitive skill categories in a more balanced way (see Figure S1, Nomis = WM game). Therefore, these four cognitive sub-domains (AF/WM, EF, MF, VSF) were chosen for the implementation of the PEMOCS concept (Table S2).

S.3.1) Personalizing the Allocation of the Cognitive Sub-dimensions

To allocate the four cognitive domains to "least" to "most" impaired [see 'D.3.1) Personalized allocation of the cognitive domains'], the MoCA [Montreal Cognitive Assessment, (Nasreddine et al. 2005)] is used. Achieved percentage values of the four domains in the MoCA at baseline are ordered; the lowest is assigned to IV, the second lowest to III, and so forth. To confirm the assignment or in case two domains rank equally, further cognitive baseline assessments are considered. For this purpose, the participant's results of a Simple reaction test (SRT, (Sturm 2006)), the N-back test (NBT, n=2 (Schellig and Schuri 2009)), the Trail-making test B (TMT-B, (Rodewald et al. 2012)), the Stroop test (Schuhfried 1999), and a Mental rotation test (MRT, (Bratfisch and Hagmann 2004)) are compared to age-matched norms (Table S2). This delivers a score between 0 and 100 for each test, which represents how well the participant performed in the test compared to the norm sample, and also categorizes them into 'below average' (< 16), 'average' (16-84), and 'above average' (> 84). By comparing the scores of the different tests, the order of the domains from "least" to "most" impaired is delivered.

Domain	MoCA Items	Further tests
Attention / Working Memory	Read digits, Read letters, Serial subtraction	SRT, NBT
Executive Functions	TMT B, Fluency, Abstraction	TMT-B, Stroop
Memory	Delayed Recall	-
Visuospatial Functions	Cube, Clock	MRT

***Supplementary Table S2**: (Nasreddine et al. 2005, Freitas et al. 2012, Coen et al. 2016). SRT: Simple reaction test, NBT: N-back test, TMT-B: Trail-making test B, Stroop: Stroop test, MRT: Mental rotation test.*

S.3.2) Assigning Motor-cognitive Tasks to the Sub-dimensions of the Extended Taxonomy

The different motor-cognitive tasks within this exergaming system are on the one hand determined by the different "Dividat Senso" games, which target different cognitive functions (Supplement 2). On the other hand, various motor tasks can be executed on the pressure sensitive plate, e.g. steps, squats, running movements and jumps (Table S3). To categorize these motor tasks, they were first assigned to one of the two sub-dimensions of 'Action Function' (Figure S1) by the following rules; 'Body stability' includes tasks, where the centre of pressure (COP) moves within stable or slightly and slowly moving limits of stability. 'Body transport', on the other hand, includes tasks, where the COP moves within constantly and rapidly moving limits of stability. Second, the motor tasks within the two sub-

dimensions (Body stability, Body transport) are ranked in ascending difficulty and allocated to three motor-task levels a, b and c (Table S3).

By combining the different games and motor tasks, different versions of all games were derived. Possible combinations were determined based on the game mechanics, e.g. some games require directed stepping and are therefore not applicable for combination with body-weight shifting. Each game version was then assigned to a motor-cognitive skill category in the extended taxonomy (Figure S1). To do so, the allocation rules presented in 'D.3.2) Assigning Motor-cognitive Tasks to the Sub-Dimensions of the Extended Taxonomy' were applied as follows:

- Stationary: games with a still game scene
  In-motion: games with a moving game scene
- No Intertrial Variability: stimuli appear in a fixed sequence and speed
  Intertrial Variability: stimuli appear in a random sequence and / or speed
- Body Stability: games, which are played with a "stability" motor task (see Table S4)
  Body Transport: games, which are played with a "transport" motor task (see Table S4)
- No Object Manipulation: participant cannot manipulate (move) the objects in the game
  Object Manipulation: participant can manipulate (move) an object in the game
- Cognitive Dimension: Main target cognitive functions of the games were grouped to the four sub-dimensions, attentional functions (AF) and working memory (WM), executive functions (EF), memory functions (MF), and visuospatial functions (VSF), see Supplement 2.

Category	Level	Tasks			
Body stability	a	BWS	Steps	Star-Steps (SS)	Steps turned
	b	BWS in squat	Cross-Steps	Squats	SS on one leg
	c	BWS on b-pad	Steps on b-pad	Squats on b-pad	SS turned
Body transport	a	Walking	Step-touches (ST)	Dribbling	Jump out, step in
	b	Walking + squat	ST crossed	Dribbling + squat	Jump on one foot
	c	Walking on toes	ST on b-pad	ST turned	Jump both feet

Supplementary Table S3*: Motor tasks in this application example of the PEMOCS concept. The tasks are assigned to "Body Stability" or "Body Transport" and categorized into motor-task levels a (easiest) to c (most difficult). For a more detailed description of the motor tasks, see Supplement 2, Table S6. BWS; body-weight shifts. B-pad; soft balance pad used as an unstable surface, which is placed on the plate.*

Action Function<br>Environmental Context	Body Stability<br>No Object Manipulation<br>A		Body Stability<br>Object Manipulation<br>B		Body Transport<br>No Object Manipulation<br>C		Body Transport<br>Object Manipulation<br>D	
Stationary<br>No Intertrial Variability<br>1	AF / WM<br>Simple_1A<br>Cloudy_1A	EF	AF / WM<br>Cloudy_1B	EF<br>Evolve_1B	AF / WM<br>Simple_1C	EF<br>Birds_1C	AF / WM	EF<br>Evolve_1D
	MF<br>Simon_1A<br>Shop_1A	VSF	MF	VSF	MF<br>Simon_1C<br>Shop_1C	VSF	MF	VSF
Stationary<br>Intertrial Variability<br>2	AF / WM<br>Simple_2A<br>Nomis_2A	EF<br>Divided_2A<br>Birds_2A<br>Flexi_2A	AF / WM<br>Cloudy_2B	EF<br>Evolve_2B	AF / WM<br>Simple_2C<br>Nomis_2C	EF<br>Divided_2C<br>Birds_2C<br>Flexi_2C	AF / WM<br>Scooper_2D	EF<br>Evolve_2D
	MF<br>Simon_2A<br>Shop_2A	VSF<br>Gears_2A	MF<br>EvoMem_2B	VSF<br>Hexa_2B<br>Tetris_2B	MF<br>Simon_2C<br>Shop_2C	VSF<br>Gears_2C	MF	VSF<br>Hexag_2D<br>Tetris_2D
In-motion<br>No Intertrial Variability<br>3	AF / WM<br>Targets_3A	EF<br>Habitats_3A	AF / WM<br>Lumina_3B	EF	AF / WM<br>Targets_3C	EF<br>Habitats_3C	AF / WM	EF
	MF	VSF<br>Gears_3A	MF	VSF<br>Hexagon_3B	MF	VSF	MF	VSF<br>Hexagon_3D
In-motion<br>Intertrial Variability<br>4	AF / WM<br>Flaneur_4A<br>Targets_4A	EF<br>Habitats_4A	AF / WM<br>Ski_4B<br>Ladybug_4B<br>Lumina_4B	EF<br>Evolve_4B	AF / WM<br>Flaneur_4C<br>Targets_4C	EF<br>Habitats_4C	AF / WM<br>Ladybug_4D<br>Lumina_4D	EF<br>Evolve_4D
	MF<br>HabiMem_4A	VSF<br>Gears_4A	MF<br>LumRuck_4B<br>EvoMem_4B	VSF<br>Hexagon_4B<br>Snake_4B<br>Tetris_4B	MF<br>HabiMem_4C	VSF<br>Gears_4C	MF<br>EvoMem_4D	VSF<br>Hexagon_4D<br>Snake_4D<br>Tetris_4D

***Supplementary Figure S1**: Extended taxonomy with an example how the cognitive domains could be allocation and the different games versions in the motor-cognitive skill categories. AF / WM: attention and working memory, EF: executive functions, MF: memory, VSF: visuospatial functions. Colours: Difficulty levels based on (Wuest et al. 2014); red / orange: level 1-2; yellow: level 3; green: level 4; blue: level 5; purple / grey: level 6-7. Game names: numbers (e.g. Simple_**1**A) represent environmental sub-dimension of the game version, letters (e.g. Simple_1**A**) the action function sub-dimension.*

S.3.3) Progression from Session to Session

The difficulty levels 1 and 2 as well as 6 and 7 were fused to provide enough variability for the training sessions as in these levels, several motor-cognitive skill categories in the extended taxonomy remained empty (Figure S1). Therefore, the following five difficulty levels exist in this application example:

- 1-2: red / orange, including motor-skill categories 1A, 1B, 2A
- 3: yellow, including motor-skill categories 1C, 2B, 3A
- 4: green, including motor-skill categories 1D, 2C, 3B, 4A
- 5: blue, including motor-skill categories 2D, 3C, 4B
- 6-7: purple / grey, including motor-skill categories 3D, 4C, 4D

All participants start in difficulty level 1-2 (first training session is standardized) and stay there for both trainings in week 1. From the second session on, progression steps to subsequent sessions are determined using the procedure presented in ‘D.3.3) Progression from session to session’. Participants rate their perceived performance and perceived motor-cognitive task difficulty using the visual analogue scales (Figure 2) in each session. As objective evaluation of challenge, the training supervisor notes their perception of how optimally challenged the participant was on a scale of -2 (strongly overchallenged) to +2 (strongly underchallenged, Figure S2). This S-score (Figure S3) represents the OP-score in this application example [see Figure 3 and ‘D.3.3) Progression from Session to Session’].

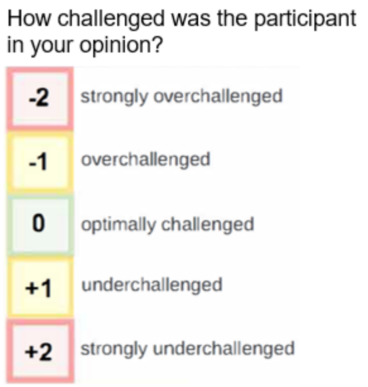

Supplementary Figure S2: *Scale for the supervisor's evaluation of the participant's challenge.*

Based on the three scales, the progression steps are determined as outlined in the following flowchart (Figure S3). The subjective ratings of the participant are transferred into challenge scores (0, +/- 1, +/- 2) based on the defined target areas (P-score PP and P-score PTD in Figure S3). These two scores are averaged to receive the mean P-score (participant challenge score). The S-score is derived from the supervisor's evaluation of the participant's challenge (Figure S3). Then, the three ratings are averaged to receive the preliminary progression steps. In case the participant and the supervisor agreed on the experienced challenge (mean P-score and S-score do not differ by 2 or more), these progression steps are then implemented to the next session. In case, however, mean P-score and S-score do differ by 2 or more, two measures are taken: On the one hand, the participant is again explained how to use the rating scales, as it is suspected that they may over- or underestimate their performance. On the other hand, the supervisor discusses the situation with the rest of the supervisor team, who decides together if the progression steps should be adapted and if yes, how (Figure S3). The final progression steps are then implemented to the next training session.

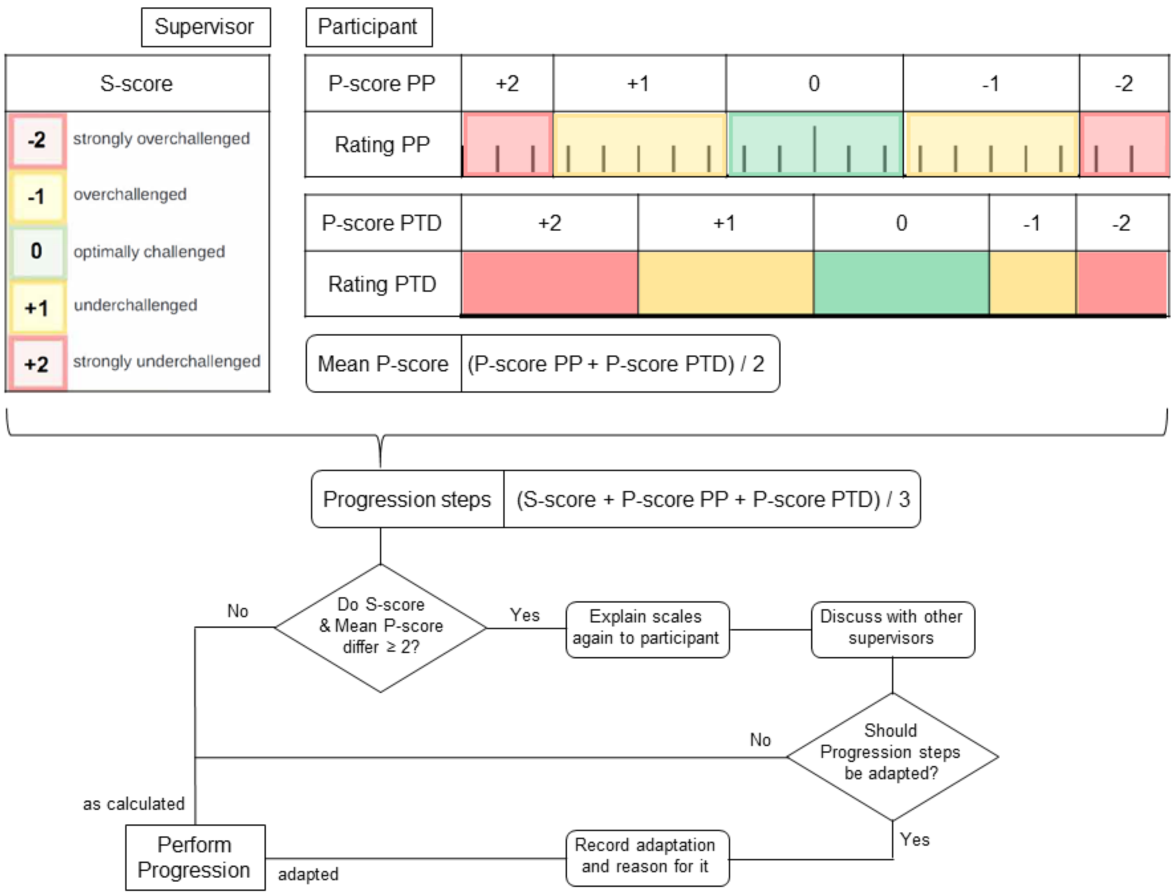

Supplementary Figure S3*: Flowchart showing procedure for personalized external progression. P-score: participant challenge score; PP: perceived performance; PTD; perceived task-difficulty; S-score: supervisor challenge score.*

Addition: Sub-levels

After defining different game versions and assigning them to the difficulty levels, still large variation in game settings and different possible motor tasks per game version were available. Therefore, to provide more variability and standardize the progression of game settings within each difficulty level, we additionally defined four sub-levels per difficulty level. Game difficulty based on settings such as speed or amount of different stimuli increases from sub-level 1 to 4. If participants stay in the same difficulty level for more than one session, they progress through the sub-levels session per session (1 → 2 → 3 → 4). However, a sub-level can be repeated in case of reasonable legitimation of the training supervisor. Generally, when starting a new difficulty level (due to pro- or retrogression), participants start in sub-level 1. However, in case a participant will probably be under-challenged with sub-level 1, the training supervisor is authorized to start with sub-level 2.

S.3.4) Progression within Sessions

The design of each session follows the PEMOCS concept, including a 3-minute warm-up, a main-part with 2-3 blocks, and a 3-minute cool-down [see 3.3.4) Progression within Sessions, Figure 3]. To

provide progression over the blocks, the motor tasks levels are used (Table S3, Figure S4). The motor tasks are applied as follows (compare Figure S4):

- Warm-up: any motor task that serves the purpose of re-familiarization
- First block: motor-task level a
- Middle block: motor-task level c
- Last block: motor-task level b (b or c if there is no middle block)
- Cool-down: any motor task that fosters a feeling of success

The progression *within* the blocks ensures the focus on the most impaired cognitive domain. Based on the session time (Table S1), the time for each block and game was defined (Table S4). A game should last between 90 and 180 seconds, therefore, in weeks 1 and 2, there are two blocks, while in weeks 3 to 12, there are three blocks. In each block, the game targeting the most impaired domain (IV) lasts 180s. The remaining time of the block is distributed equally to the games targeting the less impaired domains (I-III, Table S4). The games are arranged in each block as defined in 'D.3.4) Progression within Sessions' (Figures 3 & S4).

**Week**	**Main-part time** [min]	**# Blocks**[a]	**Time per block** [min]	**Game duration**[b] [s] most impaired	other domains
1-2	24	2	12	180	180
3-4	26	3	8 2/3	180	115
5-6	28	3	9 1/3	180	127
7-8	30	3	10	180	140
9-10	32	3	10 2/3	180	154
11-12	34	3	11 1/3	180	167

Supplementary Table S4: *Time main-part and training blocks. [a] #Blocks determined by training time, so that no game lasts longer than 180s. [b] most impaired domain out of AF, EF, MF, and VSF determined from baseline assessments, see 'Personalized allocation of the cognitive domains'.*

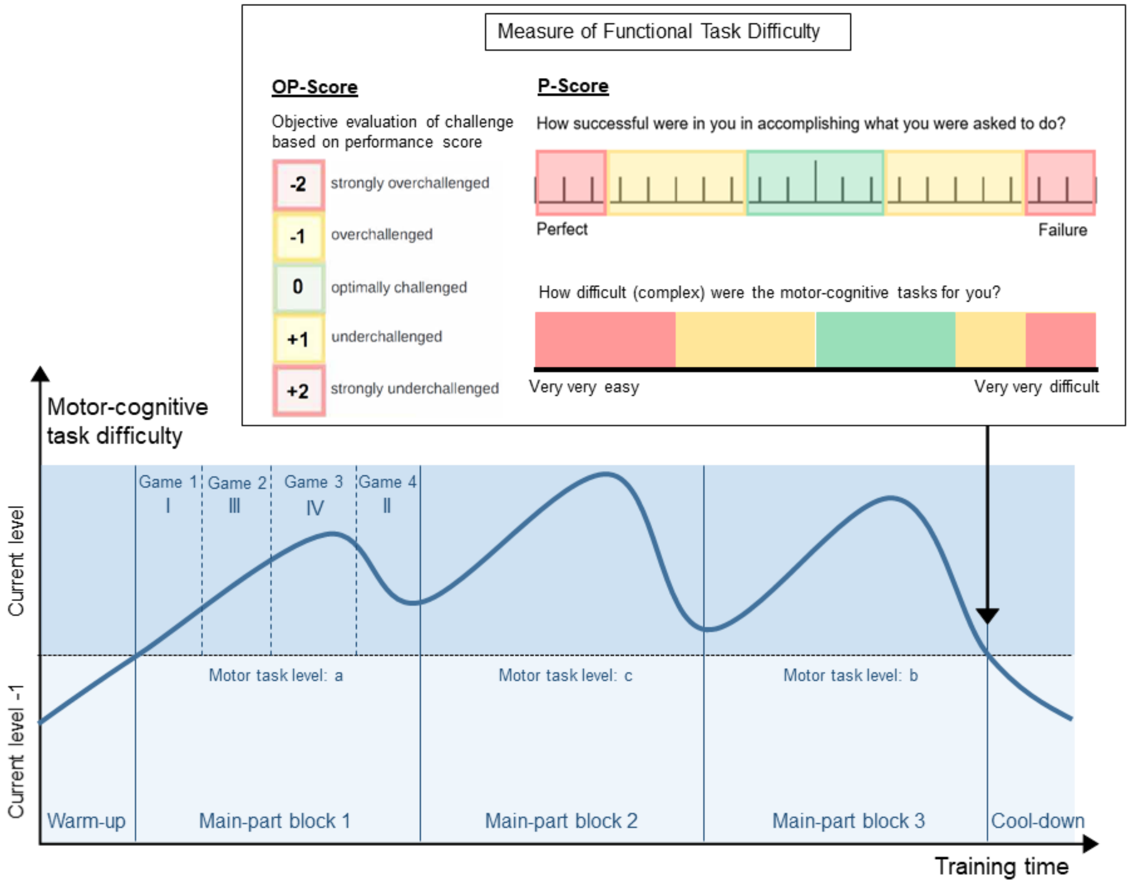

Supplementary Figure S4: *Design of each training session with warm-up, three blocks in the main-part, and cool-down. Each block contains 4 games (as shown in block 1). To achieve the shown curve of motor-cognitive task difficulty, each of the three blocks includes motor tasks of one of the three motor task levels (a: least difficult, c: most difficult) and the four games in each block target one of the four cognitive domains (domain I – least impaired domain; domain IV – most impaired domain). The supervisor's and participant's ratings of challenge are collected after the last of block of the main-part.*

S.4) Variability Rules

Variability is provided in three ways in the application example of the PEMOCS concept. (1) The different "Dividat Senso" games are allocated to the corresponding motor-cognitive skill categories of the extended taxonomy. This ensures that the participants are exposed to new games every time they progress to a new difficulty level. (2) Most games are available in different versions; for instance without or with a moving scene or inter-trial variability, providing additional variability. (3) The games are variably combined with different motor tasks in the levels, which provides variability in the resulting motor-cognitive tasks. To ensure that variability are put into practice, the following variability rules were defined:

- Each game from the current difficulty level should be played at least once a week.
- Each motor task in the current difficulty level should be performed at least once a week.
- Provide different games and motor tasks in the different blocks, if possible.

- In case a game should be played > 2x / session (e.g. because it is the only one of a domain in the current sub-level), it is allowed to replace it once by another game from a lower difficulty level.
- In level 6-7, the warm-up and cool-down games can also be chosen from difficulty level 4 (instead only from difficulty level 5).
- For participants, who stay in level 6-7 for at least 4 sessions and cannot progress further, two games in two different domains per session can be exchanged with one of the following games from sublevel 4.4: Nomis_2C, Flexi_2C, Shop_2C, Simon_2C. Game settings at least as difficult as in sublevel 4.4 should be used, e.g. longer sequences or shopping lists or Flexi_2C can be complemented with an additional calculation task.
- Besides these rules, game preferences by the participants can be considered.

Refining Steps after the Feasibility Study

Besides the steps, which are described in the main document, two further problems had been detected in the feasibility study, which directly relate to the application as presented here. (1) Several cognitive functions, namely working memory, memory, and visuospatial functions were under-represented in the list of motor-cognitive activities that were provided by the exergaming system "Dividat Senso" (Huber et al. 2021). Therefore, several new games were developed for the upcoming RCT. This helped ensuring the ability to focus on all of the tailored cognitive domains, and filling more motor-cognitive skill categories [see 'E.3) Fill empty motor-cognitive skill categories']. (2) We found in the feasibility study (Huber et al. 2021), that the motor-cognitive task difficulty had been below the targeted range optimal for motor learning and training in chronic stroke (Akizuki and Ohashi 2015). We, therefore, integrated new motor tasks, new games and game settings with higher nominal task difficulty into the concept (Table S2, for a more detailed description of the motor tasks, see Supplement 2). With the larger selection of motor-cognitive tasks, especially with higher nominal task difficulty, the PEMOCS concept should now provide sufficient functional task difficulty and intensity for a wide range of chronic stroke survivors.

Limitations

The presented application example of the PEMOCS concept renders several limitations. First, the applied training variables range at the lower border of the presented recommendations [compare 'D.2) Training Dosage']. However, as this application example will be used in a clinical trial, it also had to fulfil feasibility criteria. Based on the experience in the feasibility study (Huber et al. 2021), we suspected that a longer study duration, a higher frequency, or longer sessions would decrease the recruitment rate of the RCT. Nevertheless, the planned training variables still move within the recommended range. (2) Despite our effort to fill as many motor-cognitive skill categories as possible, several still remained empty (Figure S1). This was due to the selection of available games, and some limitations in the possibility to adjust game mechanics. We accounted for this by fusing the levels 1 and 2, as well as 6 and 7 [section 'S.3.3) Progression from Session to Session'], by introducing the sub-levels, and by adding additional variability rules for the levels with many empty categories [section 'S.4) Variability Rules']. (3) The objective evaluation of challenge in this application example is the supervisor's perception of how strongly the participant was challenged. This is not as objective as it should optimally be (compare 'Limitations and Future directions' in the main article). Optimal would be, if the exergaming system would deliver one or several performance parameters, which would allow the determination of whether the participant has reached a performance threshold that implies a progression or retrogression to the next level. This was not possible with the used system, and no other

performance parameter was yet available (see 'Future Directions and Limitations' in the main article). We mentioned the need for research on feasible performance parameters already in our feasibility study (Huber et al. 2021). Unfortunately, this was not possible in the time since, as more research on different performance parameters and their indication for optimal functional task difficulty is needed. Therefore, supervisor's ratings will be used. Subjectivity of the rating is reduced by instructions for the supervisors on how to evaluate the performance of the participant as objectively as possible, and by them noting their evaluation without knowing that the participant's rating will be.

References

Akizuki, K. and Y. Ohashi (2015). "Measurement of functional task difficulty during motor learning: What level of difficulty corresponds to the optimal challenge point?" Hum Mov Sci **43**: 107-117. doi: 10.1016/j.humov.2015.07.007.

Altorfer, P., M. Adcock, E. D. de Bruin, F. Graf and E. Giannouli (2021). "Feasibility of Cognitive-Motor Exergames in Geriatric Inpatient Rehabilitation: A Pilot Randomized Controlled Study." Frontiers in Aging Neuroscience **13**: 842. doi: 10.3389/fnagi.2021.739948.

Bratfisch, O. and E. Hagmann (2004) "Vienna test system - Visual awarness (3D) - Manual.".

Coen, R. F., D. A. Robertson, R. A. Kenny and B. L. King-Kallimanis (2016). "Strengths and Limitations of the MoCA for Assessing Cognitive Functioning: Findings From a Large Representative Sample of Irish Older Adults." J Geriatr Psychiatry Neurol **29**(1): 18-24. doi: 10.1177/0891988715598236.

Freitas, S., M. R. Simoes, J. Maroco, L. Alves and I. Santana (2012). "Construct Validity of the Montreal Cognitive Assessment (MoCA)." J Int Neuropsychol Soc **18**(2): 242-250. doi: 10.1017/S1355617711001573.

Huber, S. K., J. P. O. Held, E. D. de Bruin and R. H. Knols (2021). "Personalized Motor-Cognitive Exergame Training in Chronic Stroke Patients—A Feasibility Study." Frontiers in Aging Neuroscience **13**(663). doi: 10.3389/fnagi.2021.730801.

Jaggi, S., A. Wachter, M. Adcock, E. D. de Bruin, J. C. Moller, D. Marks, . . . E. Giannouli (2023). "Feasibility and effects of cognitive-motor exergames on fall risk factors in typical and atypical Parkinson's inpatients: a randomized controlled pilot study." Eur J Med Res **28**(1): 30. doi: 10.1186/s40001-022-00963-x.

Manser, P., L. Michels, A. Schmidt, F. Barinka and E. D. de Bruin (2023). "Effectiveness of an Individualized Exergame-Based Motor-Cognitive Training Concept Targeted to Improve Cognitive Functioning in Older Adults With Mild Neurocognitive Disorder: Study Protocol for a Randomized Controlled Trial." JMIR Res Protoc **12**: e41173. doi: 10.2196/41173.

Manser, P., H. Poikonen and E. D. de Bruin (2023). "Feasibility, usability, and acceptance of "Brain-IT"-A newly developed exergame-based training concept for the secondary prevention of mild neurocognitive disorder: a pilot randomized controlled trial." Front Aging Neurosci **15**: 1163388. doi: 10.3389/fnagi.2023.1163388.

Nasreddine, Z. S., N. A. Phillips, V. Bedirian, S. Charbonneau, V. Whitehead, I. Collin, . . . H. Chertkow (2005). "The Montreal Cognitive Assessment, MoCA: a brief screening tool for mild cognitive impairment." J Am Geriatr Soc **53**(4): 695-699. doi: 10.1111/j.1532-5415.2005.53221.x.

Rodewald, K., M. Weisbrod and A. S. (2012) "Vienna test system - Trail making test - Langensteinbacher version (TMT-L) - Manual.".

Schättin, A., S. Häfliger, A. Meyer, B. Früh, S. Böckler, Y. Hungerbühler, . . . U. Götz (2021). "Design and evaluation of user-centered exergames for patients with multiple sclerosis: multilevel usability and feasibility studies." JMIR serious games **9**(2): e22826.

Schellig, D. and U. Schuri (2009) "Vienna test system - N-Back verbal (NBV) - Manual.".

Schuhfried, G. (1999) "Vienna test system - Stroop Interence test (Stroop) - Manual.".

Seinsche, J., E. D. de Bruin, I. Carpinella, M. Ferrarin, S. Moza, F. Rizzo, . . . E. Giannouli (2022). "Older adults' needs and requirements for a comprehensive exergame-based telerehabilitation system: A focus group study." Front Public Health **10**: 1076149. doi: 10.3389/fpubh.2022.1076149.

Seinsche, J., E. D. de Bruin, E. Saibene, F. Rizzo, I. Carpinella, M. Ferrarin, . . . E. Giannouli (2023). "Feasibility and Effectiveness of a Personalized Home-Based Motor-Cognitive Training Program in Community-Dwelling Older Adults: Protocol for a Pragmatic Pilot Randomized Controlled Trial." JMIR Res Protoc **12**: e49377. doi: 10.2196/49377.

Sturm, W. (2006) "Vienna test system - Perceptional and attentional functions: Alertness (WAFA) - Manual. ."

Swanenburg, J., F. Buchi, D. Straumann, K. P. Weber and E. D. de Bruin (2020). "Exergaming With Integrated Head Turn Tasks Improves Compensatory Saccade Pattern in Some Patients With Chronic Peripheral Unilateral Vestibular Hypofunction." Front Neurol **11**: 601. doi: 10.3389/fneur.2020.00601.

Swanenburg, J., K. Wild, D. Straumann and E. D. de Bruin (2018). "Exergaming in a Moving Virtual World to Train Vestibular Functions and Gait; a Proof-of-Concept-Study With Older Adults." Front Physiol **9**(988): 988. doi: 10.3389/fphys.2018.00988.

Swinnen, N., M. Vandenbulcke, E. D. de Bruin, R. Akkerman, B. Stubbs, J. Firth and D. Vancampfort (2021). "The efficacy of exergaming in people with major neurocognitive disorder residing in long-term care facilities: a pilot randomized controlled trial." Alzheimers Res Ther **13**(1): 70. doi: 10.1186/s13195-021-00806-7.

Wuest, S., R. van de Langenberg and E. D. de Bruin (2014). "Design considerations for a theory-driven exergame-based rehabilitation program to improve walking of persons with stroke." Eur Rev Aging Phys Act **11**(2): 119-129. doi: 10.1007/s11556-013-0136-6.
