# Supplementary material for: PEMOCS: theory derivation of a concept for PErsonalized MOtor-Cognitive exergame training in chronic Stroke—a methodological paper with an application example
Source: Front Sports Act Living. 2024 Jun 10;6:1397949. doi: 10.3389/fspor.2024.1397949 (PMC11194322; doi:10.3389/fspor.2024.1397949)
Supplement: Supplementary file 2 [file Datasheet2.pdf]

Supplement 2: Descriptions of Games and Motor Tasks in the Application Example of the PEMOCS concept

Table S5: Description of Games, Game Versions, and Motor Tasks

Colour code: black - existing games and settings; green - innovations in games and settings developed in the course of the PEMOCS project and related projects in our lab

Motor tasks: The exergame system provides feedback to the direction and timing of the motor response. Type (e.g. if a step or a step-touch is to be executed) and quality (e.g. if the participant executes the squat low enough) of the PEMOCS-specific motor tasks (Table S3) are instructed and feedbacked by the training supervisor.

**Game**	**Cognitive Domain & Function(s)**	**Task Description**	**Game Versions**	**Settings**<br>* *orally instructed settings, which require no adjustment in the software settings*	**Motor Tasks**<br>→ *orally instructed and feedbacked by the training supervisor*
**Birds**<br>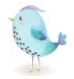	Executive Functions (EF)<br>Reasoning, Problem solving	Four objects (birds, other animals, flowers) appear in the four directions and a feather in the centre. Assign the feather to the same coloured bird by stepping into the according direction as quickly as possible.	Birds_1C<br>Birds_2A<br>Birds_2C	fixed speed, only one direction<br>fixed or random speed, random sequence<br>id.	any transport task except any walking<br>any stability task except any BWS<br>any transport task except any walking
**Cloudy**<br>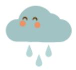	Attentional Functions (AF)<br>Sustained attention	Three flowers grow in pot and have to be watered. A cloud floats over the pots and waters them by rain. By bodyweight shifts, control the position of the cloud to water all pots.	Cloudy_1A<br>Cloudy_1B<br>Cloudy_2B	water 1 flower*<br>water flowers in a fixed sequence*<br>water flowers randomly*, bonus*	any BWS, keep COP still<br>any BWS<br>any BWS
**Divided**<br>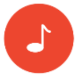	Executive Functions (EF)<br>Task switching, Alertness	Four white circles are shown on the screen. One of them changes its colour to red. React as quickly as possible by stepping in the according direction. Sometimes, a higher or a deeper tone appears instead of a red circle. Step forward as reaction to the higher, and a backward as reaction to the deeper tone.	Divided_2A<br>Divided_2C	fixed or random speed, random sequence<br>id.	any stability task except any BWS<br>any transport task except any walking
**Evolve**<br>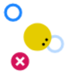	Executive Functions (EF)<br>Planning, selective attention, visual scanning	A yellow eater, blue target rings, and red obstacle crosses appear, and move in different speeds over the whole screen. The eater can be moved by body-weight shifts or walking on the plate. Collect rings, while avoiding obstacles.	Evolve_1B<br>Evolve_1D<br>Evolve_2B<br>Evolve_2D<br>Evolve_4B<br>Evolve_4D	targets do not move, no obstacles, move eater in a constant rhythm from left to right*<br>targets and obstacles do not move<br>id.<br>targets and obstacles move<br>id.	any BWS<br>any walking<br>any BWS<br>any walking<br>any BWS<br>any walking

Supplementary Material:

PEMOCS: Theory derivation of a concept for PErsonalized MOtor-Cognitive Training in chronic Stroke

**EvoMem**<br>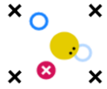	Memory Functions (MF)<br>Short-term memory<br>Planning, selective attention, visual scanning	A number of objects is "placed" on different locations on the screen, e.g. in the four corners, and their location has to be remembered. The instructor names one object at a time, and the eater has to be moved to the according location. While doing so, targets should be collected and obstacles avoided.	EvoMem_2B<br>EvoMem_4B<br>EvoMem_4C	targets and obstacles do not move<br>targets and obstacles move<br>id.	any BWS<br>any BWS<br>any walking
**Flaneur**<br>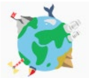	Attentional Functions (AF)<br>Sustained attention	The view of a stroll is shown on the screen. The speed of walking is determined by the cadence of steps performed on the plate. While walking, name passing objects that belong to a defined category (e.g. all blue objects).	Flaneur_4A<br>Flaneur_4C	-	steps on the spot<br>any walking or dribbling, walk around
**Flexi**<br>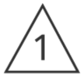	Executive Functions (EF)<br>Processing speed and task switching	A: A number is seen in the middle of the screen and four others around it. Step into the direction of the next higher number as quickly as possible.<br>B: Additionally, either a circle or a triangle appears around the number in the centre. Step to the next higher number and alternate between circles and triangles.	Flexi_2A<br>Flexi_2C	fixed speed, random sequence<br>id.	any stability task except any BWS<br>any transport task expect any walking
**Gears**<br>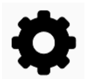	Visuospatial Functions (VSF)<br>Mental rotation	A train with gears appears in the centre, and four different traces in the four directions. Step into the direction of the trace, which matches the gear.	Gears_2A<br>Gears_2C<br>Gears_3A<br>Gears_4A<br>Gears_4C	fixed or random speed, random sequence, gears not moving<br>fixed speed, only 2 directions, gears moving<br>fixed or random speed, random sequence, gears moving	any stability task except any BWS<br>any transport task expect any walking<br>any stability task except any BWS<br>any transport task expect any walking<br>id.
**Habitats**<br>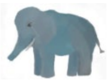	Executive Functions (EF)<br>Inhibitory control, Alertness	Animals move through the four habitats on the screen. If an animal appears in an unusual habitat, step into this direction to catch it. Make sure not to disturb animals in their proper habitat.	Habitats_3A<br>Habitats_3C<br>Habitats_4A<br>Habitats_4C	fixed speed, only 2 directions<br>id.<br>fixed or random speed, random sequence<br>id.	any stability task except any BWS<br>any transport task expect any walking<br>any stability task except any BWS<br>any transport task expect any walking
**HabiMem**<br>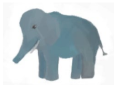 	Memory Functions (MF)<br>Short-term memory, Inhibitory control, Alertness	While playing Habitats, repeat the animals that appeared after a defined number, e.g. after every four animals.	HabiMem_4A<br>HabiMem_4C	fixed or random speed, random sequence, 3-5 animals*	any stability task except any BWS<br>any transport task expect any walking

**Hexagon**<br>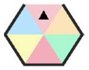	Visuospatial Functions (VSF)<br>Spatial perception, Mental rotation	An arrow is presented in a moving environment of hexagons with one open edge each. Step to the left or right to turn the hexagons in order to pass the arrow through the open edges.	Hexagon_2B<br>Hexagon_2D<br>Hexagon_3B<br>Hexagon_3D<br>Hexagon_4B<br>Hexagon_4D	very very slow speed, random sequence, long way out*<br>fixed, normal speed, defined sequence<br>id.<br>fixed, normal speed, random sequence<br>id.	any stability task except any BWS<br>any transport task expect any walking<br>any stability task except any BWS<br>any transport task expect any walking<br>any stability task except any BWS<br>any transport task expect any walking
**Ladybug**<br>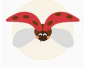	Attentional Functions (AF)<br>Sustained attention, Selective attention	Navigate the ladybug shifting your body-weight to the left and right so to collect as many flowers as possible. Avoid flying into a stone.	Ladybug_4B<br>Ladybug_4D	-	any BWS<br>any walking
**Lumina**<br>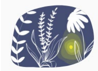	Attentional Functions (AF)<br>Sustained attention	Navigate the firefly by shifting your bodyweight into all directions. Try to collect lights, if you want.	Lumina_3B<br>Lumina_4B<br>Lumina_4D	constantly move the firefly L-R*<br>randomly move the firefly into all directions*, try to catch lights*	any BWS<br>any BWS<br>any walking
**LumRuck**<br>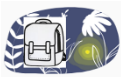	Memory Functions (MF)<br>Short-term memory, Sustained attention	While playing Lumina, play the game "I'm going on a trip and I'm taking…" (in German: "I pack into my backpack…") with the supervisor. Name one or two objects at a time.	LumRuck_4B	randomly move the firefly into all directions*, try to catch lights*	any BWS
**Nomis**<br>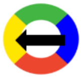	Working Memory (WM)	A series of tones with corresponding colour lights is presented. Inversely repeat the tone series by making steps to the field with the right colour.	Nomis_2A<br>Nomis_2C	3 tones or more, all directions<br>id.	any stability task except any BWS<br>any transport task expect any walking
**Scooper**<br>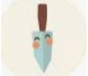	Attentional Functions (AF)<br>Sustained attention, Alertness	Carrots appear in different locations in the garden on the screen. Step precisely on each carrot and go into a squat to harvest it.	Scooper_2D	-	any walking
**Shopping Tour**<br>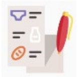	Memory Functions (MF)<br>Short-term memory	Remember the shopping list, which is first presented to you. Then look at the appearing groceries and items, and collect things that were on the shopping list by stepping to right, while leaving things that were not on the shopping list by stepping to the left.	Shop_1A<br>Shop_1C<br>Shop_2A<br>Shop_2C	only 1 item<br>id.<br>several items<br>id.	any stability task except any BWS<br>any transport task expect any walking<br>any stability task except any BWS<br>any transport task expect any walking
**Simon**<br>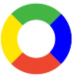	Memory Functions (MF)<br>Short-term memory	A series of tones with corresponding colour lights is presented. Repeat the tone series by making steps to the field with the right colour.	Simon_1A<br>Simon_1C<br>Simon_2A<br>Simon_2C	1-2 tones, only 2 directions<br>id.<br>3 tones or more, all directions<br>id.	any stability task except any BWS<br>any transport task expect any walking<br>any stability task except any BWS<br>any transport task expect any walking

**Simple** 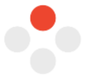	Attentional Functions (AF)<br>Alertness	There are four white circles on the screen. One of them changes its colour to red. React as quickly as possible by making a step in the direction of the red circle.	Simple_1A<br>Simple_1C<br>Simple_2A<br>Simple_2C	fixed speed, fixed sequence<br>id.<br>fixed or random speed, random sequence<br>id.	any stability task except any BWS<br>any transport task expect any walking<br>any stability task except any BWS<br>any transport task expect any walking
**Ski** 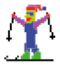	Attentional Functions (AF)<br>Sustained attention, Visual scanning	Navigate the skier by shifting your body-weight to the right and left. Avoid trees, rocks and snow monsters.	Ski_4B	-	any BWS
**Snake** 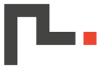	Visuospatial Functions (VSF)<br>Spatial perception	A snake sneaks across the screen. Help the snake to eat the red squares through navigating it with steps to the right, left, front or back.	Snake_4B<br>Snake_4D	-	any stability task except any BWS<br>any transport task expect any walking
**Targets** 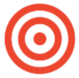	Attentional Functions (AF)<br>Sustained attention, Planning	Four targets are seen on the screen. From all directions, balls appear and fly in the direction of one of the targets. React with a step in the direction of the target as soon as the ball reaches the centre of the target.	Targets_3A<br>Targets_3C<br>Targets_4A<br>Targets_4C	fixed speed, only 2 directions (L-R or F-B)<br>id.<br>fixed or random speed, random sequence<br>id.	any stability task except any BWS<br>any transport task expect any walking<br>any stability task except any BWS<br>any transport task expect any walking
**Tetris** 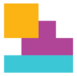	Visuospatial Functions (VSF)<br>Mental Rotation, Spatial perception	Stones in different shapes fall from the top of the screen. The stones can be rotated by 90 degrees with steps forward and moved sideways with steps to the left or right. Create horizontal lines of ten units without gaps at the bottom of the screen. Full lines will disappear and still incomplete lines will drop. Hinder the lines from growing and filling the screen.	Tetris_2B<br>Tetris_2D<br>Tetris_4B<br>Tetris_4D	very very slow speed<br>id.<br>fixed, normal speed<br>id.	any stability task except any BWS<br>any transport task expect any walking<br>any stability task except any BWS<br>any transport task expect any walking

Legend: Overview and description of all Dividat Senso games and game versions (compare Figure S1). Regarding Game Versions: numbers stand for the environmental sub-dimensions, and letters for the action function sub-dimensions. Regarding Settings: id. stands for ‘the same as above’. Setting descriptions going over two lines refer to both game versions on these lines. Regarding Motor Tasks: ‘any’ stands for the information that any motor task belonging to this sub-dimension / category can be chosen (compare Table S6).
